# Traumatic Glaucoma Managed With Ab Interno Trabeculotomy (Trabectome®): A Case Report

**DOI:** 10.7759/cureus.83670

**Published:** 2025-05-07

**Authors:** Alejandro M Perez, Naomi E Gutkind, Ji Liu, Tomas M Grippo

**Affiliations:** 1 Ophthalmology, Bascom Palmer Eye Institute, Miami, USA; 2 Ophthalmology and Visual Science, Yale School of Medicine, New Haven, USA; 3 Ophthalmology, Centro Oftalmológico Grippo, Buenos Aires, ARG

**Keywords:** ab interno, angle recession glaucoma, blunt trauma, trabectome, trabecular meshwork

## Abstract

A 55-year-old patient sustained a bungee cord injury to his left eye, resulting in a traumatic cataract, vitreous prolapse into the anterior chamber, and extensive angle recession. Despite cataract surgery with pars plana vitrectomy and maximal medical therapy, intraocular pressure (IOP) remained elevated at 40 mmHg. The patient was diagnosed with mixed-mechanism traumatic glaucoma. Ab interno trabeculotomy with the Trabectome® system (MicroSurgical Technology, Redmond, WA, USA) successfully lowered IOP over 12 months, significantly reducing the medication burden. To the best of our knowledge, this is the first published textual case report describing the use of Trabectome® in this setting. In this case, ab interno trabeculotomy proved safe and effective in managing complex traumatic glaucoma.

## Introduction

Ab interno trabeculotomy with the Trabectome® system (MicroSurgical Technology, Redmond, WA, USA) is a minimally invasive glaucoma surgery (MIGS) designed to target the trabecular meshwork (TM) and enhance aqueous humor outflow [1]. This internal approach to surgical glaucoma procedure minimizes tissue trauma, offers a high safety profile, and allows for rapid recovery with minimal impact on quality of life [1,2]. The Trabectome® employs high-frequency microelectrocautery, combined with infusion and aspiration, to selectively ablate and remove TM [2-4]. Blood reflux into the anterior chamber, caused by an inversion of the pressure gradient from Schlemm’s canal during ablation, typically resolves spontaneously without long-term effects [1,5]. A meta-analysis showed that Trabectome® reduces intraocular pressure (IOP) by about 31%, lowering it to around 15 mmHg, and can reduce medication burden [2,6,7].

Angle recession glaucoma (ARG) is a difficult-to-treat entity, in which trauma to the iridocorneal angle leads to scarring and dysfunction of the aqueous outflow pathways. Effective treatment strategies for ARG remain limited, as ARG is often considered a risk factor for surgical failure in conventional glaucoma surgery [8]. One study reported a trabeculectomy success rate of 43% in ARG patients compared to 74% in those with primary open-angle glaucoma (POAG) [8,9]. Alternatives such as laser trabeculoplasty have demonstrated limited success in treating ARG [10]. MIGS may play a role in early disease. A recent case report demonstrated the successful management of ARG using gonioscopy-assisted transluminal trabeculotomy (GATT) [11]. In this case report, we describe the use of ab interno trabeculotomy with the Trabectome® system as a surgical approach for managing traumatic mixed-mechanism glaucoma with extensive angle recession (AR), highlighting its potential role in this challenging clinical setting. Although a similar technique has been demonstrated in an instructional video by Nils Loewen [12], to the best of our knowledge, this is the first published textual case report detailing the clinical course, surgical rationale, and outcomes using Trabectome® in this context.

## Case presentation

**Figure 1:**
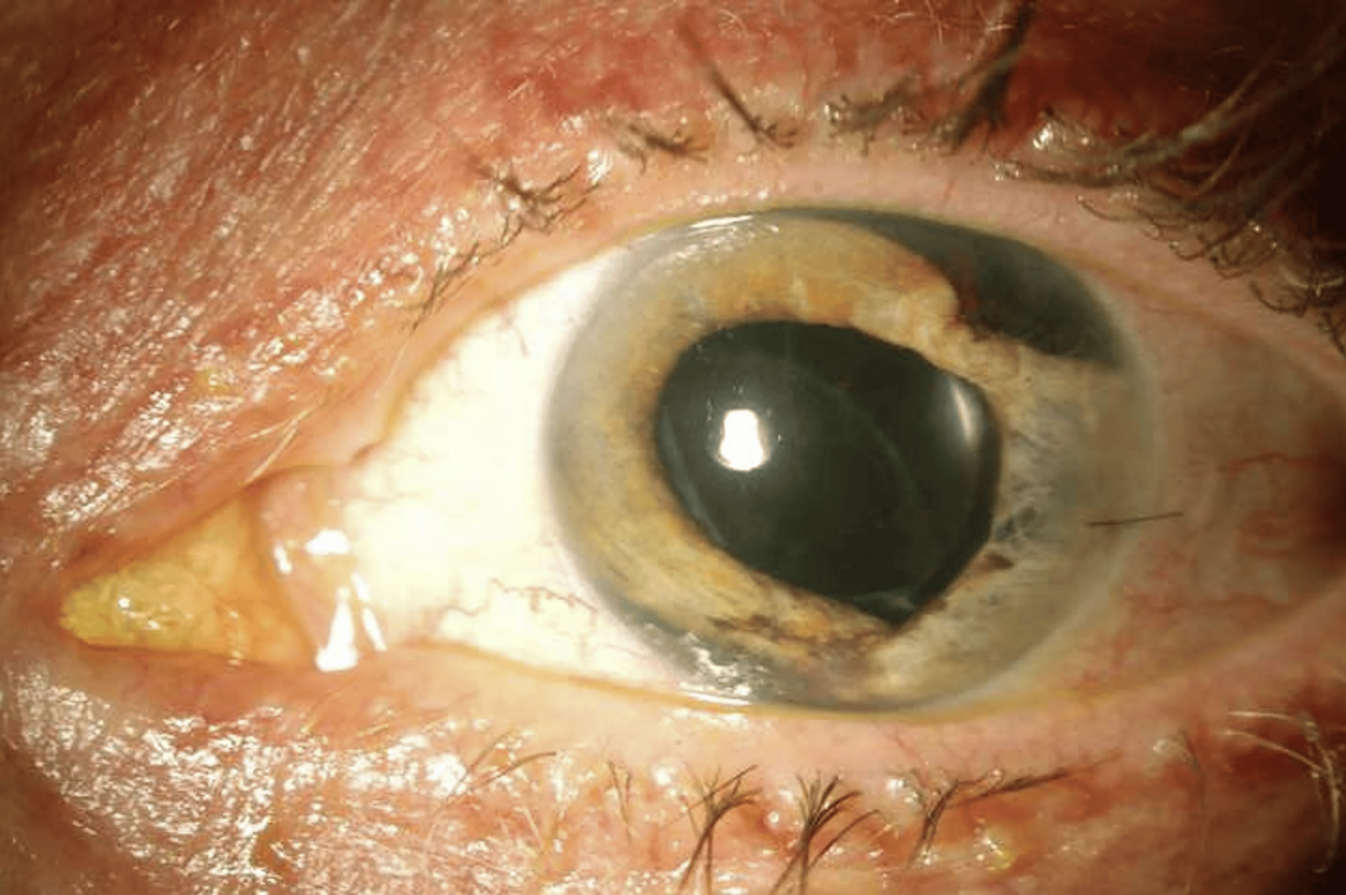
External photograph of left eye. Note superotemporal and inferonasal iridodialyses, surgical pupil and centered posterior intraocular lens.

A 55-year-old male patient was referred for elevated IOP following blunt trauma from a bungee cord two weeks prior. At initial presentation, the patient had mild corneal edema, a superotemporal and inferonasal iridodialysis, a traumatic cataract with vitreous prolapse into the anterior chamber, and vitreous hemorrhage (Figure 1). Under the care of the referring provider, he was initiated on a comprehensive regimen for IOP control, which included dorzolamide-timolol, bimatoprost, brimonidine, loteprednol, nepafenac, atropine, and oral acetazolamide. Despite this, his IOP remained above 30 mmHg. He underwent a combined cataract extraction, intraocular lens implantation, and pars plana vitrectomy (PPV) with a capsular support ring placement. Postoperatively, his visual acuity improved to 20/40; however, his IOP continued to exceed 40 mmHg despite his ongoing medical regimen. While the elevated IOP was initially attributed to angle recession, the clinical picture suggested a mixed-mechanism traumatic glaucoma. Postoperative inflammation, pigment deposition, vitreous prolapse obstructing the trabecular meshwork, altered aqueous dynamics following PPV, and a possible steroid-induced pressure response likely contributed to persistent IOP elevation. These overlapping mechanisms compounded the outflow obstruction from the extensive-angle recession.

**Figure 2:**
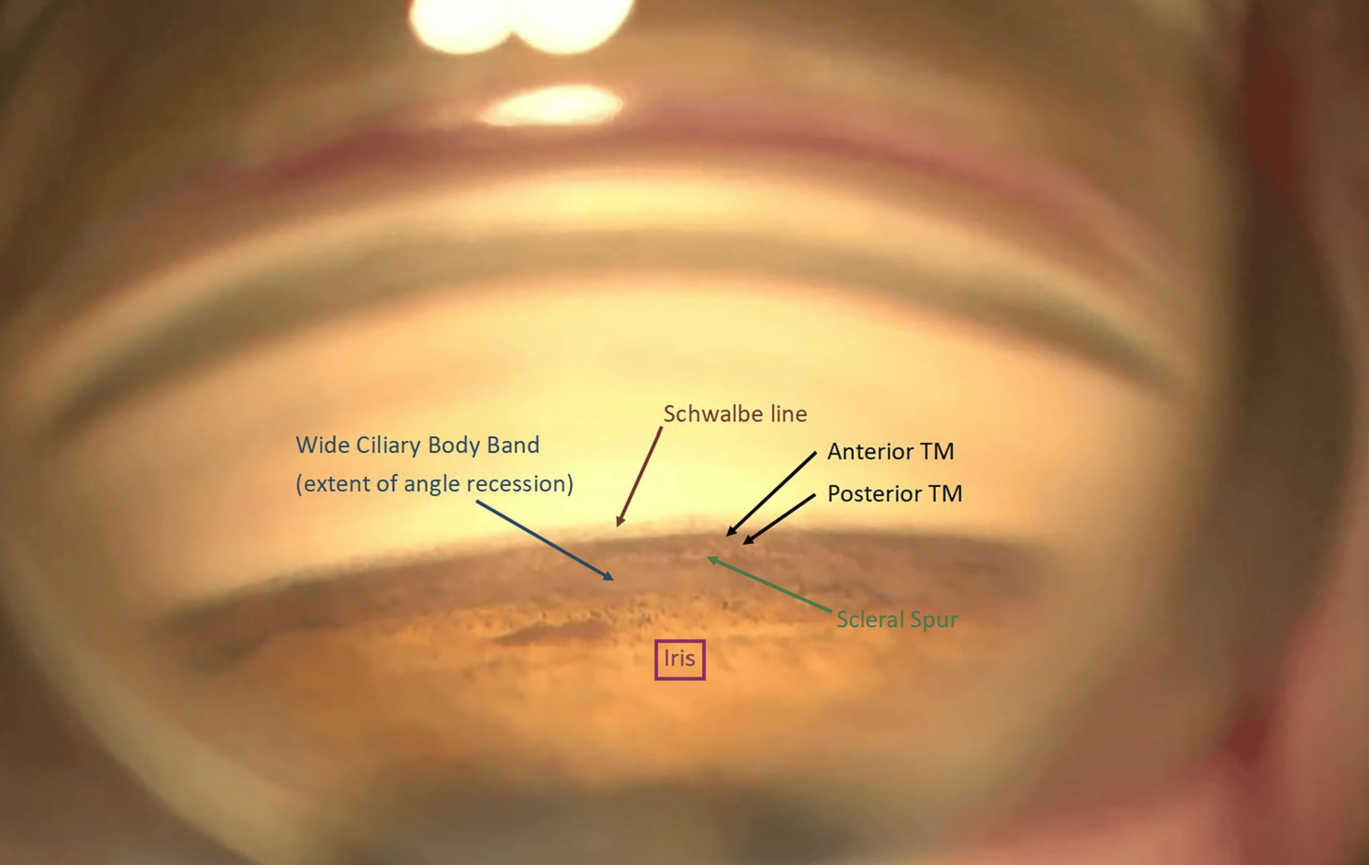
Gonioscopic view of the nasal angle demonstrating key anatomical landmarks from anterior to posterior. Schwalbe’s line (brown arrow) marks the anterior limit of the angle, followed by the anterior and posterior trabecular meshwork (black arrows), with increased pigmentation posteriorly. The scleral spur is identified (green arrow), and a widened ciliary body band (blue arrow) reflects the extent of angle recession. The recessed or posteriorized iris is noted inferiorly (purple box), consistent with traumatic angle recession. TM: trabecular meshwork

Gonioscopy showed 270 degrees of AR with no peripheral anterior synechiae (Figure 2).

**Figure 3:**
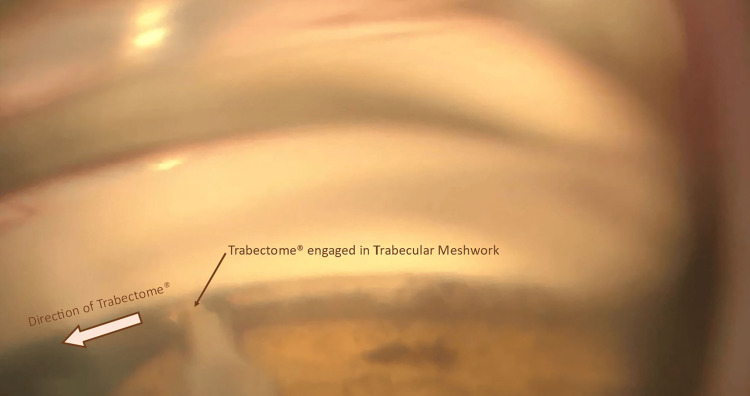
Intraoperative gonioscopic view showing the Trabectome® engaged within the trabecular meshwork. The ablation direction is indicated by the arrow, with visible removal of pigmented trabecular meshwork along the treatment path.

The posterior segment exam was unremarkable, with a cup-to-disc ratio of 0.3 bilaterally and an intact retinal nerve fiber layer on optical coherence tomography. A Humphrey visual field testing was normal. Given the uncontrolled IOP despite maximal tolerated medical therapy, a decision was made to proceed with ablation of the trabecular meshwork using the Trabectome® system to prevent glaucomatous damage. After the creation of a sterile field in the operating room, a 1 mm paracentesis was performed with a microvitreoretinal blade. A clear corneal incision of 1.7 mm was then created with a slit knife on the temporal cornea. The Trabectome® handpiece was inserted through the main incision into the eye, where its tip engaged the collapsed TM, creating space to position it within Schlemm's canal between the TM and the inner and outer walls of the canal. A TM ablation of about 120 degrees was then achieved (Figure 3).

A surgical video (Video 1) demonstrates the ablation technique in real-time, highlighting the extent of TM removal and visualization of Schlemm’s canal under gonioscopic guidance.

**Video 1:** Surgical video demonstrating ab interno trabeculotomy with the Trabectome® system. Gonioscopic visualization captures the complete ablation of the trabecular meshwork, establishing a direct outflow pathway into Schlemm’s canal.

On post-op day one, IOP was controlled at 15 mmHg. He had a 0.5 mm hyphema and was continued on prednisolone acetate, moxifloxacin, and pilocarpine. The hyphema cleared in one week, and the prednisolone was tapered over four weeks. The pilocarpine was tapered over four months. IOP remained in the mid-teens throughout the first year of follow-up on dorzolamide-timolol combination drops twice daily without further complications. A summary of the patient’s clinical course, intraocular pressure trends, and treatment adjustments is provided in Table 1.

**Table 1:** Clinical timeline and IOP summary. IOP: intraocular pressure; POD: postoperative day; POM: postoperative month; CEIOL: cataract extraction with intraocular lens implantation; PPV: pars plana vitrectomy.

Date/event	Clinical note	IOP (mmHg)	Medications
Day 0 – injury	Blunt trauma, traumatic cataract, angle recession	30s	Maximal therapy
Day 14 – initial surgery	CEIOL + PPV	30s	Maximal therapy
Day 30 – second surgery	Ablation of the trabecular meshwork using the Trabectome® system	40s	Maximal therapy
POD 1	Immediate post-op status post Trabectome®	15	Prednisolone, pilocarpine
POM 1	IOP stable, hyphema resolved	14-16	Prednisolone tapered, pilocarpine
POM 3	IOP stable, medications reduced	14-16	Dorzolamide-timolol
POM 12	IOP stable, no complications	14-16	Dorzolamide-timolol

## Discussion

Traumatic glaucoma can result from multiple mechanisms, including inflammation, TM injury, hyphema, AR, ghost cells, secondary angle closure, and lens trauma [13]. Clinical features such as elevated baseline IOP, trabecular pigmentation, hyphema, iridodialysis, iris sphincter tears, lens displacement, and extensive-angle recession have been associated with an increased risk of glaucoma development [8,14]. In our patient, the acute IOP elevation following blunt trauma was likely multifactorial. Although initial management with cataract extraction and PPV addressed the traumatic cataract and vitreous hemorrhage, IOP remained uncontrolled. The primary mechanism was likely TM dysfunction secondary to extensive AR. Still, additional contributors included trabecular obstruction, postoperative inflammation from PPV, and possible steroid-induced pressure response. Taken together, these overlapping factors suggest a diagnosis of mixed-mechanism traumatic glaucoma rather than isolated ARG. Importantly, studies show that 7-9% of patients with AR involving more than 180° eventually develop glaucoma, highlighting the need for lifelong monitoring for late-onset disease [8,15].

Given the patient's elevated IOP >40 mmHg despite maximally tolerated medical therapy, the surgical team considered multiple options, including trabeculectomy, glaucoma drainage devices (GDDs), and MIGS. Trabectome® was selected as a conjunctiva-sparing approach that offers a high safety profile and preserves tissue for future surgical interventions if needed [1]. Compared to GDDs, which carry a higher risk of hypotony and are typically reserved for more advanced or refractory cases, and trabeculectomy, which has lower success rates in post-traumatic glaucoma, Trabectome® was chosen for its minimally invasive nature, effective IOP-lowering potential, and safety advantages [9]. This decision was also informed by the patient's relatively preserved optic nerve and visual field, suggesting early glaucomatous risk without current structural or functional damage.

Histopathologically, AR involves a laceration between the longitudinal and circular muscle fibers of the ciliary body, causing posterior displacement of the circular muscles, along with the iris root, and disruption of the TM [2,16,17]. Early in the injury, IOP may be normal or low due to ciliary body hyposecretion, cyclodialysis clefts, or trabecular tears that create direct communication between Schlemm’s canal and the anterior chamber [8]. Over time, degenerative changes in the TM, such as atrophy, fibrosis, and hyalinization develop, impairing aqueous outflow and resulting in elevated IOP [8,18].

Initial management of ARG typically involves medical therapy [8]. Laser trabeculoplasty is normally ineffective [10,19]. Contact transscleral diode laser cyclophotocoagulation has mostly been described in advanced or refractory stages of ARG [20].

In cases that require surgery for IOP control, ARG is a known risk factor for surgical failure. GDDs, particularly Molteno implants (Nova Eye Medical, Fremont, CA, USA), have shown some success but are less effective in ARG than cases with previous failed filtering procedures or in aphakic or pseudophakic eyes [21]. Trabeculectomy with mitomycin C has effectively controlled IOP in ARG, outperforming trabeculectomy without antimetabolites and Molteno implants [22,23]. However, overall success rates for filtering surgeries in ARG are lower compared to POAG, and they carry significant risks of postoperative complications, such as wound leaks, shallow or flat anterior chambers, hypotony, infection, choroidal effusion, and hemorrhage [24].

MIGS may offer moderate and prompt IOP control with a better safety profile than traditional filtering surgeries [2,25]. Additionally, preservation of conjunctival tissue allows for future filtering procedures, if necessary. The best MIGS for ARG is still under investigation. A recent report of GATT in an ARG patient showed success [11]. GATT, which shears the TM, is considered more traumatic and poses higher risks of hyphema compared to Trabectome® [26]. Additionally, the Trabectome® has the advantage of selectively removing compromised TM, including areas of dense pigmentation, peripheral anterior synechiae, fibrotic scars, hyalinized trabeculae, and posterior extension of Descemet’s membrane, thereby restoring aqueous outflow by facilitating direct communication with Schlemm’s canal [1]. Further studies are needed to compare the efficacy of different MIGS in ARG.

This case adds to the limited literature on surgical management of traumatic glaucoma with mixed mechanisms, offering a detailed account of Trabectome® use in a scenario that involved angle recession, post-vitrectomy inflammation, and possible steroid-induced IOP elevation. While a similar technique was visually demonstrated by Nils Loewen [12], to the best of our knowledge, no peer-reviewed case report has previously described the clinical decision-making and postoperative course in this setting.

## Conclusions

This case highlights the potential role of Trabectome® in managing mixed-mechanism traumatic glaucoma involving extensive angle recession. The procedure resulted in effective IOP control and medication reduction for at least one year. Further research is warranted to compare surgical options and evaluate long-term outcomes in this challenging subset of glaucoma.
